# The Beneficial Effects of Saffron Extract on Potential Oxidative Stress in Cardiovascular Diseases

**DOI:** 10.1155/2021/6699821

**Published:** 2021-01-19

**Authors:** Xin Su, Chao Yuan, Li Wang, Runqi Chen, Xiangying Li, Yijun Zhang, Can Liu, Xu Liu, Wanping Liang, Yanwei Xing

**Affiliations:** ^1^Guang'anmen Hospital, China Academy of Chinese Medical Sciences, Beijing 100053, China; ^2^Dezhou Second People's Hospital, Dezhou 253000, China; ^3^Xingtai People's Hospital, Xingtai 054001, China; ^4^Shanxi Province Cancer Hospital, Shanxi Medical University, Taiyuan 030000, China; ^5^The First Affiliated Hospital, Hebei North University, Zhangjiakou 075000, China; ^6^School of Acupuncture-Moxibustion and Tuina, Beijing University of Chinese Medicine, Beijing 100029, China

## Abstract

Saffron is commonly used in traditional medicines and precious perfumes. It contains pharmacologically active compounds with notably potent antioxidant activity. Saffron has a variety of active components, including crocin, crocetin, and safranal. Oxidative stress plays an important role in many cardiovascular diseases, and its uncontrolled chain reaction is related to myocardial injury. Numerous studies have confirmed that saffron exact exhibits protective effects on the myocardium and might be beneficial in the treatment of cardiovascular disease. In view of the role of oxidative stress in cardiovascular disease, people have shown considerable interest in the potential role of saffron extract as a treatment for a range of cardiovascular diseases. This review analyzed the use of saffron in the treatment of cardiovascular diseases through antioxidant stress from four aspects: antiatherosclerosis, antimyocardial ischemia, anti-ischemia reperfusion injury, and improvement in drug-induced cardiotoxicity, particularly anthracycline-induced. Although data is limited in humans with only two clinically relevant studies, the results of preclinical studies regarding the antioxidant stress effects of saffron are promising and warrant further research in clinical trials. This review summarized the protective effect of saffron in cardiovascular diseases and drug-induced cardiotoxicity. It will facilitate pharmacological research and development and promote utilization of saffron.

## 1. Introduction

Crocin, crocetin, and safranal, which are active components of saffron, have been widely studied. Saffron (*Crocus sativus* L.) is a perennial flowering plant of the family Iridaceae [1, 2]. Its officinal part is obtained from the dried stigma, which is a rare spice worldwide, and it is used in the treatment of several diseases [3, 4]. Saffron is widely cultivated in Iran and southern European countries, such as Greece, and it is rarely cultivated in China [5]. Saffron was first documented in the “Collected Essentials of Species of Materia Medica” in China, even though it was used earlier than the 1500s. Its uses have been recorded during the time of the Assyrians [6]. The main active compound of saffron is crocin [7]. Crocin, a saffron glycoside, is a water-soluble carotenoid having predominantly four analogues: crocin 1, crocin 2 [8, 9], crocin 3 [10], and crocin 4 [11]. In traditional Chinese medicine, saffron is considered to promote blood circulation, remove blood stasis, and relieve pain [12]. Scientific studies have shown saffron to possess therapeutic value with multiple pharmacological effects, including antioxidant [13, 14], anti-inflammatory [15], and cardioprotective effects [16]. Additionally, some studies have shown that it confers protective effects against cardiovascular diseases [17], asthma [18], diabetes mellitus [19], Parkinson's disease [20], depression [21], cancer [22], and other diseases.

**Figure 1:**
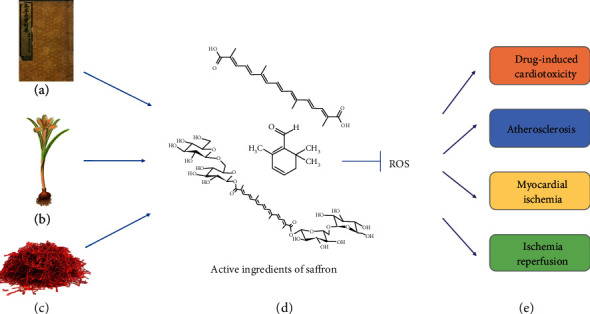
Source and functions and of saffron extract. (a) “Collected Essentials of Species of Materia Medica.” (b) Complete morphology of saffron. (c) Prepared officinal part of saffron. (d) Chemical structures of saffron compounds. (e) Pharmacological effects of saffron. Saffron was first documented in (a) “Collected Essentials of Species of Materia Medica” in China. (d) The chemical structure of saffron extract from top to bottom is crocetin, safranal, and crocin. Many studies have demonstrated that saffron compounds can protect the cardiovascular system by modulating oxidative stress and improving drug-induced cardiotoxicity. Furthermore, they have been known to have antiatherosclerosis, antimyocardial ischemia, anti-ischemia reperfusion injury, and several other effects.

Cardiovascular diseases (CVDs) are the major causes of mortality worldwide and currently provide a considerable challenge for clinical treatments [23].CVDs were the primary cause of 17.7 million deaths worldwide in 2015, which is predicted to increase to 23.6 million by 2030 [24, 25]. In recent years, the introduction of novel drugs and innovative devices has contributed to protecting patients with cardiopathy from CVDs. Recent in vitro and in vivo studies have indicated that saffron might have cardioprotective effects by modulating oxidative stress, thereby decreasing injury to the myocardium, which protects the heart (see Figure 1). Attention has been drawn to the widespread medicinal value of saffron, and researchers have focused on its potential bioactive components and mechanisms of action, particularly antioxidant stress, anti-inflammatory, and cardioprotective effects. Few general reviews have discussed the phytochemical constituents and cardioprotective pharmacological attributes of saffron to bridge the gap among the studies in recent years. This review summarizes the protective effects of saffron compounds in CVDs and chemotherapy-induced cardiotoxicity to facilitate pharmacological research, development, and utilization of saffron.

## 2. Review

### 2.1. Chemistry and Bioactivity of Saffron Compounds

**Figure 2:**
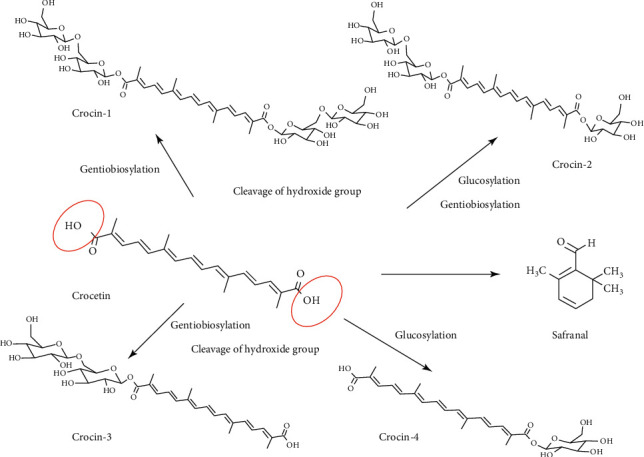
Chemical structure of active components of saffron. These components are crocin, crocetin and safranal, in which crocin has four analogues: crocin 1, crocin 2, crocin 3, and crocin 4.

Crocin is distributed in flowers, fruits, stigmas, leaves, and roots of saffron, and the content of crocin in different parts varies. For example, crocin is predominantly distributed in the stigma. Crocin is a group of hydrophilic carotenoids that are either mono- or di-glycosylpolyene-esters of crocetin in which D-glucose and/or D-gentiobiose occur as carbohydrate residues [26]. The aglycone (crocetin) of these natural products is the same (see Figure 2). Additionally, safranal is also the main active compound in saffron. The crocin family includes various glycosyl esters, of which four types have been detected in saffron. Crocin analogues, including crocin 1-4, are predominantly glycosides of *trans*-crocetin in saffron, among which experimental research on crocin 1 is more popular [27]. All crocin derivatives, except crocin-1, have been reported to be *cis*-trans isomer pairs [28]. Other than the three derived compounds (crocin, crocetin, and safranal), a number of carotenoid compounds have been identified in saffron, including minor amounts of lycopene, alpha/beta carotene, zeaxanthin, phytoene, and phytofluene, which are oil-soluble color pigments of saffron [29]. In the 1940s, a study indicated that *trans*-crocin underwent photoisomerization reactions and converted to *cis*-crocin using high-performance liquid chromatography (HPLC) [30]. The yield of the saffron stigma is extremely low. The extraction process depends on agricultural and environmental conditions in the area of the plant's origin. The stigma accounts for 7.4% of the total flower mass; hence, to obtain a kilogram of dried stigma requires over 150,000 flowers [31]. The laborious and hand-made production of saffron and its low yield account for its reputation as the ultimate high-cost spice and an expensive traditional medicine. The quality and extraction method of saffron compounds affects the content and efficacy of crocin. At present, the common qualitative and quantitative analysis methods mainly include HPLC [32], ultraviolet-visible spectrophotometry [33], gas chromatography-tandem mass spectrometry [34], and spectroscopy [35]. Yield of saffron compounds is optimized by the solvent, temperature, light, and stirring time used in the process. Renowned for its antioxidant activity and cardioprotective effects, saffron extract is generally regarded as a water-soluble adjunct; it is soluble in diethyl ether and ethanol, as well.

### 2.2. Molecular Mechanisms of Oxidative Stress in Cardiovascular Diseases

**Figure 3:**
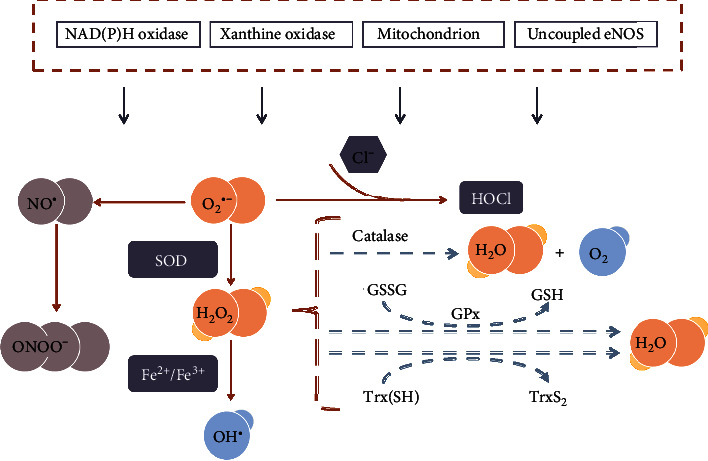
Reactive oxygen species (ROS) generation in cardiovascular diseases. NAD(P)H: nicotinamide adenine dinucleotide (phosphate); eNOS: endothelial nitric oxide synthase; NO^●^: nitric oxide; O_2_^●−^: superoxide; HOCl: hypochlorite; SOD: superoxide dismutase activity; H_2_O_2_: hydrogen peroxide; ONOO^−^: peroxynitrite; OH^●^: hydroxyl radicals; GSH: glutathione; GSSG: oxidized glutathione; GPx: glutathione peroxidase; Trx: thioredoxin. O_2_^●−^ can be generated in extracellular myocardium by NAD(P)H, uncoupled eNOS, xanthine oxidase, and mitochondrial respiration chains. H_2_O_2_ can be spontaneously converted to OH^●−^ by Fe^2+^/Fe^3+^ reaction and SOD. H_2_O_2_ can detoxify H_2_O and O_2_ by GSH peroxidase, Trx peroxidase, and catalase. Additionally, the uncoupling of eNOS decreases NO^●^ production in endothelial cells, which is further aggravated by the reduced expression and activity of eNOS. Myeloperoxidase uses hydrogen peroxide to oxidize chloride to form a strong oxidant (HOCl).

Reactive oxygen species (ROS), a group of small reactive molecules, play critical roles in the regulation of biological processes and various vital activities. ROS plays a crucial role in many CVDs, and its uncontrolled production is involved in myocardial damage. The endogenous antioxidant function as checkpoints to avoid these adverse effects of ROS, and an imbalance in the oxidant/antioxidant mechanisms leads to oxidative stress [36]. The main sources of ROS in the body are shown in Figure 3 [37–40]: (1) nicotinamide adenine dinucleotide (NADH) oxidase, (2) xanthine oxidase, (3) endothelial nitric oxide synthase (eNOS), and (4) myeloperoxidase. These pathways may be related to the development of adverse cardiovascular changes. NADH oxidase is the most important enzyme in the formation of vascular ROS [41]. ROS induce endothelial apoptosis and the expression of adhesion molecules in endothelial cells, promote lipid peroxidation, and cause the development of atherosclerosis. Atherosclerosis is caused when ROS damage the endothelial-dependent vascular function, induce endothelial apoptosis and the expression of adhesion molecules in endothelial cells, promote the proliferation and migration of vascular smooth muscle cells, and induce lipid peroxidation [42]. There are four main mechanisms for the increase in ROS in ischemic heart disease. First, after myocardial ischemia and hypoxia, the energy supply of myocardial cells is insufficient, and the levels of adenosine triphosphate degradation products (xanthine and hypoxanthine) increase, thereby generating a large amount of ROS in the process of the xanthine metabolism during its transformation into uric acid [43]. Second, a large amount of catecholamine secretion and utilization increases the synthesis, and secretion of proinflammatory cytokines or directly stimulates endothelial cells, which eventually leads to the activation of the NADH/nicotinamide adenine dinucleotide phosphate (NADPH) oxidation system and the production of ROS in cardiomyocytes. Third, the enzyme activity of the mitochondrial respiratory chain system decreases during ischemia, thereby blocking electron transfer when the energy was deficient, which changes the oxidative phosphorylation process from oxygen to H_2_O by four-electron oxidation into single-electron reduction [44, 45]. Fourth, there are obstacles in ROS clearance during ischemia, causing the accumulation of a large amount of ROS in cells, which begins the cycle of damage once again.

### 2.3. Safety of Saffron Extract in Animals and Humans

The safety of any drug used for treatment needs to be carefully evaluated to ensure that the potential side effects of treatment are clear. Although saffron has been used through the centuries as a medicinal plant, most have been performed in animals, whereas only small-scale clinical trials have been reported in recent years. Bahmani et al. [46] observed the safety of water extract of saffron and demonstrated that the oral lethal dose(LD50) is 4120 ± 556 mg/kg in mice. Ramadan et al. [47] demonstrated that the oral administration of the ethanolic extract of saffron in doses up to 5 g/kg did not cause any demonstrable acute toxic effect or death in mice, whereas Mohajeri et al. [48] found that the intraperitoneal median LD50 of ethanolic extract was 3.5 g/kg in rats. The toxic doses when saffron extract is administered intraperitoneally are lower than those studies in the oral administration. It is inaccurate to directly convert animal doses to humans because it does not take into account the different physiological functions of organisms [49]. Researchers have suggested conversion methods based on the body surface area as a guide [50]. In this case, taking the study of Bahmani et al. as an example, the equivalent human dose after conversion is 20 g for a 60 kg person in oral [51].In addition, few studies have directly evaluated the safety of saffron extract. Mohamadpour et al. [52] found that in healthy volunteers, the oral administration of crocin at a dose of 20 mg/day within one month did not produce any clinically significant adverse events compared with placebo.

### 2.4. Effect of Saffron Extract on Cardiovascular Diseases

Cell and tissue damage caused by oxidative stress are broadly implicated in human pathophysiology, particularly CVDs [53]. To date, our increasingly complex knowledge of redox signaling in the cardiovascular system has not resulted in our ability to target it for the prevention and management of CVDs. Oxidation-reduction reactions play a crucial part in physiological signal transduction, mediating receptor-coupled regulation of ion channels and transporters, kinases, and other intracellular signaling pathways in cardiomyocytes through reversible redox modification of proteins. However, there is an unbalanced change from the incipient stage of the disease process, with oxidation-reduction overwhelming cellular protective mechanisms, such as the early pathological process of atherosclerosis [54–56]. In the case of hyperglycemia and abnormal glucose metabolism, glucose is oxidized by itself or enters the mitochondria of cells through the polyol pathway. This process induces the production of excess oxygen free radicals, which exceed the ability of endothelial cells to protect themselves and cause endothelial damage. This occurs in the majority of cardiovascular pathologies, whether the arterial wall contributing to atherosclerosis and vascular dysfunction or the myocardium from the beginning of heart failure [57, 58]. Although a massive global burden is ascribed to CVDs, progress in the field has lagged behind others such as oncology in the development and clinical translation of effective new drugs [59]. This central role of dysregulation represents an excellent opportunity for emerging clinical therapies aimed at all levels of oxidation-reduction reactions to prevent the progression and clinical consequences of the full spectrum of CVDs. It is noteworthy that crocin, a common active component in natural medicines, may play a role in modulating CVDs through oxidation-reduction reactions. The main findings of some studies that investigated the potential antioxidative stress activity of saffron in CVDs are summarized in Table 1.

### 2.5. Antiatherosclerosis

As the dominant cause of CVDs, oxidative stress plays a vital role in atherosclerosis. In the early stage of atherogenesis, inflammation in the innermost layer of the blood vessels leads to the release of proinflammatory cytokines, adhesion molecules, and chemokines [60]. Additionally, redox stress is involved in several different representations of CVDs. The overproduction of ROS leads to inflammation and is associated with atherogenesis through several key enzymes, including nitric oxide synthase (NOS), xanthine oxidase, and NADPH oxidases [61–63]. Moreover, due to its high oxidative metabolism and low antioxidant defense, which is different from other tissues, the myocardium is susceptible to oxidative damage [64, 65].

#### 2.5.1. In Vitro Study

He et al. [17] used oxidatively modified low-density lipoprotein (Ox-LDL) to incubate bovine aortic endothelial cells (BAECs) to simulate the atherosclerotic state in vitro. After incubation with Ox-LDL, the activity of nitric oxide (NO) in culture media and that of NOS in endothelial cells were measured. Compared with the control group, crocin reduced malondialdehyde (MDA) levels and inhibited the decrease of NO. Tang et al. [66] used crocetin as an experimental drug to intervene in the same model and obtained similar results. In BAECs, Ox-LDL treatment decreased NO production and downregulated activity and mRNA expression of eNOS, which was inhibited by cotreatment with crocetin (0.1, 1, and 10 *μ*mol/L) in a dose-dependent manner.

#### 2.5.2. In Vivo Study

Zheng et al. [67] studied the pharmacological effect of crocetin on atherosclerosis in rabbits. The results showed severe atherosclerosis and hypercholesterolemia in rabbits that were fed a high-fat diet. In contrast, no severe atherosclerosis was detected in rabbits with atherosclerosis treated with crocetin, which indicated that crocetin has an antiatherosclerotic effect. The underlying mechanism thereof may be reduced activation of nuclear factor kappa B (NF-*κ*B) and inhibition of the expression of vascular cell adhesion molecule-1 (VCAM-1). In a follow-up study [68], supplementation with crocetin reduced the progression of atherosclerotic lesions and plasma levels of Ox-LDL. Moreover, crocetin increased the plasma total antioxidant capacity (by the ferric reducing/antioxidant power test) and superoxide dismutase activity (SOD) in rabbits, thereby inhibiting LDL oxidation and partially contributing to the reduction of atherosclerosis. He et al. [69] observed the influence of crocetin on experimental atherosclerosis in quails that were fed a hyperlipidemic diet. Crocetin was administered by oral gavage. The study found that crocetin inhibited the formation of aortic plaque observed using hematoxylin-eosin staining. Additionally, it reduced MDA and inhibited the decrease in NO levels in serum. Endothelial dysfunction strongly contributes to the initiation and progression of atherosclerosis, as seen in a study [66] that formed an endothelial dysfunction model by feeding a high cholesterol diet (HCD) to rabbits and measured their endothelium-dependent relaxation (EDR) evoked by acetylcholine (Ach) and thoracic aorta. The results indicated that the EDR in HCD-only fed rabbits was notably impaired, and that the maximal relaxation induced by Ach was 54% compared with that in control rabbits who were fed a regular diet. Oral supplementation with crocetin (15 and 30 mg/kg) dose-dependently improved this impairment and restored the maximal relaxation to 68% and 80% compared with the control group. Supplementation with crocetin simultaneously increased the NO serum level, upregulated vessel activity, mRNA expression levels of eNOS, and vessel cyclic guanosine monophosphate content compared with those in rabbits fed with HCD alone.

#### 2.5.3. Clinical Findings

A single, short-duration trial [70] investigated the bioactivity of saffron in CVD. Oral consumption of saffron (50 mg/day for six weeks) dissolved in milk was administered to 10 healthy participants and 10 with coronary artery disease, who were compared to 10 control patients consuming milk only. The sensitivity to lipoprotein oxidation was notably reduced in both intervention groups (from 76.0 to 48.8 units in patients with coronary artery disease), whereas it was not in the control patients. Recently, a randomized, placebo-controlled clinical trial [71] that evaluated the potential impacts of saffron aqueous extract (SAE) and crocin on some atherosclerosis-related gene expression and serum levels of Ox-LDL and monocyte chemoattractant protein 1 (MCP-1) in patients with coronary artery disease (CAD). Patients were categorized into three groups: group 1 received crocin (30 mg/day), group 2 SAE (30 mg/day), and group 3 placebo for eight weeks. Lectin-like oxidized LDL receptor 1 (LOX-1), nuclear factor kappa B (NF-*κ*B), and MCP-1 in peripheral blood mononuclear cells were assessed. Compared with the placebo group, the expression of the *LOX-1* and *NF-κB* genes decreased. Serum Ox-LDL levels decreased in the crocin group after treatment, and serum MCP-1 levels decreased in the crocin and SAE groups. Crocin may have beneficial effects on patients with CAD by decreasing the expression of *LOX-1* and *NF-κB*.

### 2.6. Antimyocardial Ischemia

Myocardial ischemia (MI) is a pathological state that cannot support the normal functioning of the heart due to decreased blood perfusion and oxygen supply and abnormal myocardial energy metabolism. Hypertension, aortic insufficiency, and coronary artery occlusion are the risk factors of MI. Isoproterenol (ISO) is commonly used to simulate MI in animal experiments [72]. Oxidative stress is a critical factor in inducing cardiovascular dysfunction as it worsens the endothelial function and leads to insufficient blood supply, which is the possible pathological basis of MI. Under normal circumstances, the rate of ROS produced by oxidative stress reactions is in balance with its clearance rate [73, 74]. The most important ROS in blood vessels is the superoxide anion (O_2_^−^), and SOD can disassociate O_2_^−^ to generate H_2_O in terms of antioxidation. Generally, MDA, SOD, and NOS are used to reflect the ROS content, which is used to evaluate the degree of oxidative stress [75–77].

#### 2.6.1. In Vivo Study

Joukar et al. [78] assessed the effects of saffron compounds on rat hearts with ISO-induced myocardial injury. The results showed that the saffron+ISO group, when compared to the ISO group, significantly increased serum levels of troponin I and reduced the glutathione peroxidase (GSHPx) activity of the heart muscle. Mehdizadeh et al. [79] chose the same model to observe the antioxidant effect of the aqueous extract of saffron and safranal. Pretreatment with aqueous extract of saffron (20, 40, 80, and 160 mg/kg) or safranal (0.025, 0.050, and 0.075 mL/kg) reduced the level of MDA as a marker of lipid peroxidation in the myocardium when compared with rats treated with ISO alone. Goyal et al. [80] investigated the effects of crocin on ISO-induced cardiotoxicity with reference to hemodynamic, antioxidant, histopathological, and ultrastructural parameters. Rats were administered crocin (5, 10, and 20 mg/kg/day) or a vehicle orally for 21 d with ISO (85 mg/kg subcutaneously at 24 h intervals) on the twentieth and twenty-first days. Compared with the ISO group, a notable improvement in the activities of SOD, catalase, and reduced glutathione (GSH) levels with a reduction in the MDA content was observed. Crocin (20 mg/kg/day) significantly inhibited oxidant derangements. Light microscopy and ultrastructural analyses were used to visualize changes in the crocin group, which were improved myocardial necrosis and edema. It was demonstrated that crocin can prevent myocardial infarction induced by ISO. Another study [81] showed that crocetin ester (CE) protected against ISO-induced acute MI through the Rho/ROCK/NF-*κ*B pathway. CE (25 and 50 mg/kg) decreased the MDA content and SOD activity. Additionally, CE ameliorated the cardiac expression of Zn-superoxide dismutase, MDA5, Rho, ROCK, p-I*κ*B, and p-NF-*κ*Bp65 in ISO-induced rats. These results indicated that saffron could maintain the redox state of cells by regulating oxidative stress, which has a cardioprotective effect in ISO-induced cardiotoxicity. Jin et al. [82] observed the effects of crocin on ISO-induced cardiotoxicity in mice by intraperitoneal injection of crocin (100 and 200 mg/kg/day for 14 d). Administration of crocin improved the morphology of the heart and caused a significant reduction in oxidative stress levels (SOD, catalase, and GSH) in ISO-induced cardiotoxicity in mice. Additionally, crocin treatment suppressed the expression of NF-*κ*B and toll-like receptor 4 (TLR4). The protective effects of crocin might be achieved by regulating the TLR4/NF-*κ* B signal transduction pathway. Xue et al. [83] found that safranal played a cardioprotective role in ISO-induced cardiotoxicity in mice. Safranal (0.025 and 0.075 mL/kg) reduced the activity of serum MDA and intracellular calcium concentration and increased the activity of serum SOD. This was a step further than previous studies; saffron extract increased the activity of SOD and intracellular calcium concentration.

### 2.7. Anti-Ischemia Reperfusion Injury

Myocardial ischemia-reperfusion (IR) injury leads to disorders of the cardiac function, tissue damage, and metabolic changes. Hearts that experience IR and coronary artery insufficiency exhibit myocardial damage and abnormal ECG conduction, malignant arrhythmia, and cardiac arrest [84]. ROS and lipid peroxidation are the causes of myocardial IR injury. The effects of crocin and crocetin on myocardial IR are similar in that they play a role in improving myocardial energy metabolism through an antioxidant effect. Previous studies [85, 86] suggested that there is a close relationship between myocardial IR injury and the production of oxygen free radicals. In patients with MI, the number of intracellular free radical scavengers is reduced, resulting in a large number of free radicals that cannot be removed in time, which plays a role in promoting cell damage and accelerating cell death.

#### 2.7.1. In Vitro Study

Dianat et al. [87] studied the effects of crocin antioxidant pretreatment on hemodynamics and infarct size against IR in isolated rat hearts. Animals were divided into a control group, an ischemia-reperfusion control group, and three treatment groups: crocin (10, 20, and 40 mg/kg), vitamin E (100 mg/kg), and a combination (crocin 40 mg/kg with vitamin E 100 mg/kg). Crocin significantly improved cardiac dysfunction and reduced infarct size in rat hearts. However, the combination of crocin (40 mg/kg) and vitamin E (100 mg/kg) had a significant improvement on hemodynamic parameters and infarct size, as well as an increase in SOD and catalase enzyme activities, and a decrease in MDA was noticed. Therefore, the protective role of crocin may be due to the stability or reinforcement of antioxidant systems [88], and crocin could be useful for the treatment or prevention of cardiac dysfunction.

#### 2.7.2. In Vivo Study

Bharti et al. [89] investigated safranal-induced myocardial protection against IR injury in rats by promoting antioxidant effects. Safranal (0.1-0.5 mL/kg/day, i.p.) dose-dependently enhanced phosphorylation of Akt/GSK-3*β*/eNOS and suppressed the IKK-*β*/NF-*κ*B protein expression in IR-challenged myocardium. Moreover, safranal normalized GSHPx, LDH, and CK-MB in IR-injured myocardium in a dose-dependent manner. Efentakis et al. [90] found that SAE improved myocardial IR injury in wild-type and ApoE (-/-) mice via the Nrf2 pathway. This in vivo study established three interventions: water for injection, SAE at a dose of 60 mg/kg/day, SAE as described above, and wortmannin at a dose of 60 *μ*g/kg bolus 15 min before reperfusion. SAE reduced the infarct size by the ratio of the ischemic area to the risk area (I/R%) in wild-type and ApoE (-/-) mice. The administration of wortmannin resulted in partial inhibition of the infarct size limitation efficacy of saffron (in wild-type and ApoE (-/-) mice). Mice receiving SAE showed increased levels of eNOS, p-Akt, p-ERK1/2, p-44/p-42, and p-GSK3*β*-Ser9 and reduced inducible nitric oxide synthase (iNOS). Saffron limits myocardial infarction in wild-type and ApoE(-/-) mice in a multifaceted manner, including activation of the Akt/eNOS/ERK1/2/GSK3-*β* pathway. A study by Jahanbakhsh et al. [91] showed that crocin had a protective effect on myocardial IR-induced arrhythmia in rats. Crocin (20 mg/kg, i.p.) protected against IR injury by increasing the activity of peroxidase, SOD, and GSH, and reducing the content of MDA. Yan et al. [92] showed that crocetin (50 mg/kg, i.p.) improved myocardial injury induced by hemorrhagic shock and resuscitation in anesthetized rats. The mechanism of action is as follows: crocetin maintained the activity of total SOD, reduced the superoxide anion or free radical, weakened the activity of iNOS, and increased the production of NO. Wang et al. [93] investigated the mechanism of crocetin in repairing myocardial injury after IR in vivo. Wistar rats were randomly divided into the sham operation, IR, and crocetin groups and administered crocetin (50 mg/kg, i.g. for 7 d) or sodium carboxymethylcellulose prior to the operation. The results affirmed that pretreatment with crocetin reduced myocardial injury and oxidative stress. In this study, it was observed that IR in rats results in the upregulation of MDA and downregulation of SOD. Additionally, the administration of crocetin increased the expression of eNOS and NO, indicating that crocetin could protect myocardial cells by inhibiting ROS production and blocking inflammatory reactions.

### 2.8. Improvement in Drug-Induced Cardiotoxicity

Drug cardiotoxicity refers to the side effects of drugs that cause myocardial damage, arrhythmia, abnormal cardiac systolic or diastolic function, and cardiac hypertrophy [94, 95]. The decrease in the left ventricular ejection fraction is also considered a cardiotoxic event [96]. Cardiovascular toxicity due to therapeutic drug use has the highest incidence and severity of adverse drug reactions in late-stage clinical development [97, 98]. Based on previous reports [99–102], the role of saffron in preventing these drug-induced cardiotoxic events may be attributed to stability or increase in the capacity of antioxidant systems.

Cardiotoxicity appears to be a major cause of drug withdrawal from the pharmaceutical market [103, 104]. Several chemotherapeutic compounds, particularly antitumor drugs, have been noted for their propensity to induce dangerous cardiac-specific side effects. Cardiac toxicity has a serious impact on the quality of life of cancer patients. Moreover, the development of toxicity may lead to the adjustment or cessation of antitumor treatment, affecting the survival of patients [105]. Common chemotherapy drugs [106–108] that cause cardiotoxicity include anthracyclines, alkylating agents, antimetabolic drugs, platinum, anticell microtubule agents, and targeted therapy drugs, predominantly anthracycline-derived [109]. The main mechanism of anthracycline-induced cardiotoxicity is the production of ROS mediated by iron and the promotion of oxidative stress in the myocardium [110, 111]. Anthracyclines chelate iron ions and trigger the generation of oxygen free radicals, particularly hydroxyl radicals, which lead to lipid peroxidation of myocardial cell membranes and damage of mitochondrial DNA [112]. Compared with other cells, anthracyclines are more likely to be present in cardiomyocytes due to their myocardial affinity, the lack of catalase in the myocardium, and its weak antioxidant activity [113]. Additionally, cardiomyocytes are rich in mitochondria, which is another source of ROS; anthracycline drugs have a high affinity for cardiolipin, which can enter mitochondria and bind cardiolipin, thereby inhibiting the respiratory chain and causing cardiac damage [114–116]. Clinical research and experimental studies [117–119] show that the majority of cardiotoxicity induced by anthracycline drugs are progressive and irreversible; in particular, their first use can easily cause heart injury. Therefore, early monitoring and active prevention of anthracycline-induced cardiotoxicity are highly important [120].

#### 2.8.1. In Vitro Study

Chahine et al. [121, 122] used the model of an isolated rabbit heart perfused in retrograde. In one set of experiments, SOD was generated by electrolysis of the perfused heart solution (3 mmol/L for 30 min) in the presence and absence of saffron extract at the optimal dose (10 *μ*g/mL). In another set, researchers perfused the heart with an anthracycline, that is 3 mmol/L doxorubicin (DOX) in the presence and absence of 10 *μ*g/ml saffron extract. SOD generated by DOX significantly affected the cardiovascular function. Saffrons perfused during electrolysis help trap SOD and significantly improve the myocardial function. Another study [123] found that the administration of saffron extract during reperfusion significantly reduced oxidative myocardial damage, although it was less effective when administered before ischemia was induced. Upon confirming the protective effects of the saffron extract on DOX-induced oxidative cytotoxicity in isolated rabbit hearts, this effect was investigated in H9c2 cardiomyocytes against IR and DOX combined toxicity. H9c2 cells were treated with simulated IR and DOX in the presence or absence of saffron extract (10 *μ*g/mL). The data indicated that treatment with saffron extract attenuated DOX-induced toxicity by increasing cell viability and attenuating LDH activity. Thus, the saffron extract had a significant protective effect against DOX-induced myocardial cell death. Chu [124] demonstrated that crocin prevented doxorubicin-induced toxicity through antioxidant stress in H9c2 cells. H9c2 cardiomyoblasts were incubated with DOX (2.0 *μ*M) after treatment with crocin (40,80 *μ*M for 120 min) or were pretreated with crocin (50 *μ*M for 120 min) before being administered DOX. The results revealed that the DOX administration elevated ROS levels (SOD, catalase, and GSH) in contrast with the no treatment group, whereas crocin pretreatment notably restrained ROS expression in DOX-treated H9c2 cells.

#### 2.8.2. In Vivo Study

Razavi et al. [99, 125] used diazinon to induce cardiac injury in rats. Administration of crocin decreased MDA in the myocardium and hypoxia inducible factor-1*α* (HIF-1*α*), which showed that crocin had an antioxidative stress effect, thus playing a pharmacological role in the treatment of CVDs. HIF-1 is the master regulator of the translational response, which regulates cellular responses to hypoxia [126]. It is a heterodimeric transcription factor including HIF-1*α* and hypoxia inducible factor-1*β* subunits [127, 128]. The alpha subunit is sensitive to oxygen [129]. HIF-1*α* may have the opposite reaction with ROS at different stages. Under the hypoxia condition, HIF-1*α* could be activated by ROS through a variety of signal pathways to protect cells from hypoxia. At this point, the hydroxylation of HIF-1*α* is blocked, which leads to the stabilization of HIF-1 and protects cells from hypoxia [130]. Under normoxic conditions, the ubiquitin/proteasome pathway could degrade HIF-1*α* via tumor suppressor protein von Hippel Lindau (VHL). VHL is one of the components of ubiquitin in E3 ligase. This process is triggered by the hydroxylation of specific prolyl residues at the HIF-*α* subunits through VHL [131]. This study found that diazinon increased the HIF-1*α* protein level. The increase of HIF-1*α* may be related to the compensation mechanism after diazinon-induced oxidative stress. Crocin alleviated this condition caused by diazinon. This is consistent with other studies: under certain conditions, many antioxidants, such as ascorbic acid and catalase, reduce the HIF-1 expression via reduction of ROS [132]. Salem et al. [101] showed that crocin reduced the level of lipid peroxidation and regulated the activity of SOD and peroxidase, indicating that crocin had a protective effect on zearalenone-induced cardiotoxicity. Razmaraii [133] conducted a study to assess the protective effect of crocin on DOX-induced cardiotoxicity in rats. The rats were divided into four groups: control, DOX (2 mg/kg/48 h for 12 d), and crocin groups that received DOX as in DOX group and crocin (20 and 40 mg/kg/24 h for 20 d) starting 4 d prior to the first DOX injection. DOX treatment resulted in cardiotoxicity manifested by decreased left ventricular systolic and diastolic pressures, ejection fraction, fractional shortening, and contractility index, as compared to those of the control group. Additionally, histopathological analysis of the heart demonstrated adverse structural changes in myocardial cells following the DOX administration. Moreover, crocin treatment significantly improved DOX-induced heart damage, structural changes in the myocardium, and ventricular function. Additionally, crocin did not affect the in vitro antitumor activity of DOX. However, the limitation of the study was that crocin had not demonstrated to improve cardiotoxicity by regulating antioxidant stress. Elsherbiny [134] evaluated that crocin protected against DOX-induced myocardial toxicity in rats. Rats received DOX (3.5 mg/kg twice weekly) for three weeks with and without daily administration of crocin (10 and 20 mg/kg, orally) for three weeks. There was a significant increase in MDA levels associated with the reduced SOD activity in myocardial homogenates in DOX-administered rats compared to those of the control group. Furthermore, there was a significant dose-dependent decrease in the level of MDA and restoration of the SOD activity in myocardial homogenates of rats treated with crocin (10 and 20 mg/kg) compared to those in the DOX group. Chu [124] identified the cardioprotective action of crocin against DOX-induced cardiotoxicity in rats. Crocin (50 and 100 mg/kg/d, i.p. for 4 d) was administered to rats, and DOX (10 mg/kg/day, i.p.) administered on the fourth day. Crocin exerted positive effects on DOX-induced ROS production and changes in oxidative stress biomarkers. Crocin significantly decreased intracellular Ca^2+^ concentration and increased mitochondrial membrane potentiation in H9c2 cells. Crocin notably inhibited DOX-mediated elevated expression of TLR2 and NF-*κ*B in the ventricular tissue. These cardioprotective effects might be closely related to the TLR2/NF-*κ*B pathway.

## 3. Conclusions

**Figure 4:**
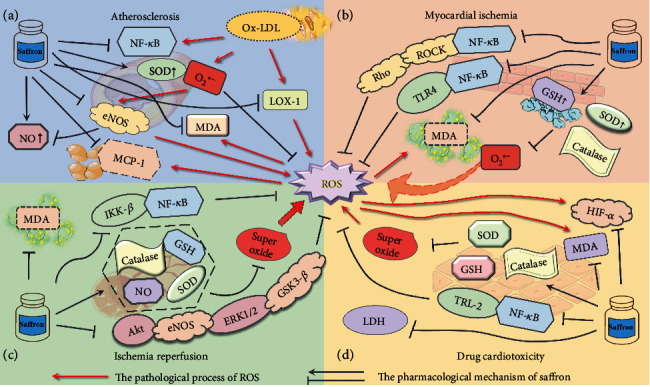
Pharmacological mechanism of crocin in the evolution of cardiovascular diseases (CVDs).ROS: reactive oxygen species; NF-*κ*B: nuclear factor kappa B; Ox-LDL: oxidized low-density lipoprotein; LOX-1: lectin-like oxidized LDL receptor 1; O_2_^●−^: superoxide; SOD: superoxide dismutase activity; MDA: malondialdehyde; eNOS: endothelial nitric oxide synthase; NO: nitric oxide; MCP-1: monocyte chemoattractant protein 1; GSH: glutathione; IKK-*β*: inhibitor of nuclear factor kappa-B kinase beta; Akt: protein kinase B; ERK 1/2: extracellular regulated protein kinases 1/2; GSK3-*β*: glycogen synthase kinase-3beta; LDH: lactate dehydrogenase; HIF-1*α*: hypoxia inducible factor-1 alpha; TLR-2: toll-like receptor 2; Rho: Ras homolog; ROCK: Rho-associated coiled-coil forming protein kinase; TLR-4: toll-like receptor 4. (a) During the evolution of atherosclerotic plaques, the main role of crocin is to reduce the oxidation of LDL and inhibit the various oxidative stress responses that ox-LDL triggers. (b) Saffron in MI regulates superoxide reaction by increasing SOD, GSH, and catalase, thereby preventing excessive myocardial injury. Saffron may regulate oxidative stress through the NF-*κ* B signaling. (c) In the progress of myocardial IR, saffron predominantly reduces the damage caused by oxidative stress by inhibiting the activation of the ROS system. (d) Saffron resists oxidative stress by regulating ROS-related enzymes and through HIF-*α*, to improve drug-induced cardiotoxicity.

Research on the use of saffron extract in CVDs has made great progress in recent years (see Figure 4). Based on the available evidence that the active components of saffron may play great role by regulating antioxidation on atherosclerosis, myocardial ischemia, ischemia reperfusion injury, and drug-induced cardiotoxicity, saffron extract can inhibit the occurrence of oxidative stress by protecting vascular endothelial and myocardial cells and normalizing the hypersensitive oxidative stress. Currently, many aspects of saffron and its bioactivity remain unknown. First, many potential mechanisms of antioxidative stress and the pharmacological effects of saffron are not clearly elucidated. The majority of the existing research on the pharmacological effects of saffron extract are relatively isolated and require more in-depth and extensive investigations. Second, the majority of the research reports on the pharmacological effects of saffron concentrate on the animal and cell level, while rarely assessing its clinical applications. Hence, it is necessary to carry out such studies to verify the potential cardioprotective efficacy of saffron extract in patients with CVDs. As a traditional medicine, saffron has been widely used since many ages in clinical practice. Therefore, it is necessary to study the effective components of saffron, such as crocin, crocetin, and safranal. Further, elucidation of the mechanisms of active constituents in saffron, its pharmacokinetics and metabolism, and the medicinal value of crocin are required for their broader application.

## Figures and Tables

**Table 1:** Detected studies reporting potential antioxidative stress effects of saffron in CVDs.

Reference	Author	Application	Component	Experiment	Model	Target
[17]	Shuying He	Atherosclerosis	Crocin	In vitro	Bovine aortic endothelial cells	MDA, NOS
[66]	F.T. Tang		Crocin	In vitro	Bovine aortic endothelial cells	NOS, eNOS
[67].	Shuguo Zheng		Crocetin	In vivo	New Zealand white rabbits	NF-*κ*B
[68]	Shuguo Zheng		Crocetin	In vivo	New Zealand white rabbits	Ox-LDL
[69]	Shuying He		Crocetin	In vivo	Quails	MDA, NOS
[66]	F.T. Tang		Crocin	In vivo	New Zealand rabbits	NOS, eNOS
[70]	S. K. Verma		Saffron	Clinical finding	Human	Ox-LDL
[71]	Nasim Abedimanesh		Saffron	Clinical finding	Human	LOX1, NF-*κ*B, MCP-1

[78]	Siyavash Joukar	Myocardial ischemia	Saffron	In vivo	Wistar rats	GSHPx
[79]	Roya Mehdizadeh		Safranal	In vivo	Wistar rats	MDA
[80]	S.N. Goyal		Crocin	In vivo	Wistar albino rats	SOD, CAT, GSH, MDA
[81]	Zhiheng Huang		Saffron	In vivo	Sprague Dawley rats	Rho/ROCK/NF-*κ*B
[82]	Weiyue Jin		Crocin	In vivo	Kunming mice	TLR4/NF-*κ*B
[83]	Yurun Xue		Safranal	In vivo	Sprague Dawley rats	MDA, SOD

[87]	Mahin Dianat	Ischemia reperfusion	Crocin	In vitro	Isolated Sprague Dawley rat hearts	SOD, CAT, MDA
[88]	Mahin Dianat		Crocin	In vitro	Isolated Sprague Dawley rat hearts	SOD, CAT, MDA
[89]	Saurabh Bharti		Crocin	In vivo	Wistar albino rats	Akt/GSK-3*β*/eNOS, GSHPx,IKK-*β*/NF-*κ*B
[90]	P. Efentakis		Saffron	In vivo	ApoE(-/-) mice	Akt/eNOS/ERK1/2/GSK3-*β*
[91]	Zahra Jahanbakhsh		Crocin	In vivo	Wistar rats	SOD, GSH, MDA
[92]	Junling Yan		Crocetin	In vivo	Sprague Dawley rats	iNOS, NO
[93]	Yanyan Wang		Crocetin	In vivo	Wistar rats	MDA, SOD, NO, eNOS

[121]	Nathalie Chahine	Drug cardiotoxicity	Saffron	In vitro	Isolated Oryctolagus cuniculus Rabbit hearts	SOD
[122]	Nathalie Chahine		Saffron	In vitro	Isolated Oryctolagus cuniculus Rabbit hearts	SOD
[123]	Nathalie Chahine		Saffron	In vitro	H9c2 cells	LDH
[124]	Xi Chu		Crocin	In vitro	H9c2 cells	SOD, GSH, catalase
[99]	Bibi Marjan Razavi		Crocin	In vivo	Wistar rats	HIF-*α*
[125]	Bibi Marjan Razavi		Crocin	In vivo	Wistar rats	MDA

[101]	Intidhar Ben Salem	Drug cardiotoxicity	Crocin	In vivo	Balb/c mice	SOD
[133]	Nasser Razmaraii		Crocin	In vivo	Wistar rats	**—**
[134]	Nehal M Elsherbiny		Crocin	In vivo	Sprague Dawley rats	MDA, SOD
[124]	Xi Chu		Crocin	In vivo	Sprague Dawley rats	TLR-2/NF-*κ*B
